# Automated Hematology Analyzers in Diagnosis of Plasmodium vivax Malaria: an Adjunct to Conventional Microscopy

**DOI:** 10.4084/MJHID.2014.034

**Published:** 2014-06-01

**Authors:** Kolakkadan Hasaf Mubeen, Clement Wilfred Devadoss, Rau Aarathi Rangan, Monnappa Gitanjali, Shetty Prasanna, VP Sunitha

**Affiliations:** Department of Pathology, M.S Ramaiah Medical College & Teaching Hospital, Bangalore. India

## Abstract

Malaria is one of the most pervasive parasitic diseases ever known to mankind affecting nearly 300 million people every year. The need for rapid diagnosis of malaria in tropical and subtropical malaria endemic areas is on the rise. In this study, we evaluated the usefulness of hematology autoanalyzers, Sysmex XE-2100 & XT-2000i in the presumptive diagnosis of malaria. Our study shows that abnormalities in WBC/BASO scattergram when combined with presence of thrombocytopenia had a high sensitivity and positive predictive value in the presumptive diagnosis of *Plasmodium vivax (P.vivax)* malaria.

## Introduction

The World Health Organization estimates that half the world’s population is at risk of malaria, with an estimated 200–300 million people developing clinical malaria every year.^1^ According to World Malaria Report (2013)nearly half (273 million) of the high-risk population outside Africa resides in India^2^ Karnataka is located in the southern peninsular region of India and was once considered as a high transmission zone for malaria but due to the implementation of rigorous control measures the malaria incidence in the state has fallen significantly. However urban malaria has continued to be a problem in the cities of Bangalore and Mangalore in Karnataka is due to the migration of people from high risk rural areas.

In 2011, 24487 malaria cases were reported in the state of Karnataka of which 21842 (89.19%) cases were infected by *Plasmodium vivax* species, indicating that *P.vivax* is the predominant species in this region. Demographic data from these cases reveal that age group 21–30 years was most affected.^3^

Light microscopy is considered as the gold standard approach in diagnosis of malaria, but it requires time and expertise. Many rapid diagnostic tests (RDT) have emerged recently to overcome these factors however these are expensive and not routinely available.^4^,^5^

As Complete Blood Count (CBC) is a baseline investigation ordered for patients with fever, there has been a growing focus on the utility of hematology analyzers in the presumptive diagnosis of malarial infection.

In the year 1999, Hancheid et al. reported the usefulness of automated hematology analyzer Cell Dyn 3500 (Abbott, Santa Clara, CA) in diagnosis of malaria even in the absence of clinical suspicion.^6^

This study was conducted to evaluate the utility of automated hematology analyzers, Sysmex XE-2100 & XT-2000i (Kobe, Japan) in the diagnosis of the malarial parasite in conjunction with peripheral smear examination, by evaluating various scattergram abnormalities.

## Materials and Methods

Routine blood samples from both outpatient and inpatient departments were analyzed during March 2013 to August 2013. Samples were collected in K2 EDTA tubes (Becton Dickinson, USA) and complete blood count analysis was done either on Sysmex XE-2100 or XT-2000i. Peripheral smear examination was done for all the cases.

Both analyzers use flow cytometry using a semiconductor laser to categorize WBCs based on the forward and side scattered light information of cells. Forward scattered light analyses the size and the side scattered light analyses the granularity of the WBCs. This data is depicted on coloured depictions namely WBC-DIFF and WBC/BASO scattergrams.

WBC-DIFF scattergram (WBC 4-part differential): RBCs are completely lysed with lysing solution “STROMATOLYSER-4DL”, at the same time this reagent acts on WBC membranes and makes them partially permeable. Following this, a fluorescent dyeing solution “STROMATOLYSER-4DS” is added to allow the fluorescent dye to enter the WBC through its permeable membranes and stain the DNA and RNA. Following this reaction, the inbuilt flow cytometer detects the forward and side scatter information based on which WBC-DIFF scattergram is obtained. By analyzing this scattergram, the analyzer gives a 4 part differential count, viz lymphocytes, monocytes, eosinophils and other granulocytes (neutrophils plus basophils).^7^

**Figure 1:**
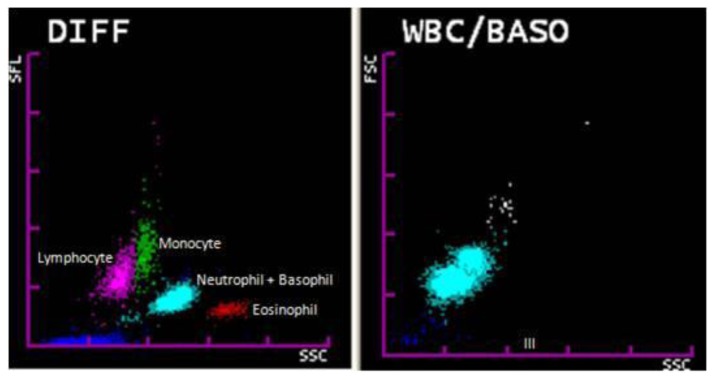
Normal WBC-DIFF and WBC/ BASO scattergram. Note area III in WBC/BASO scattergram.

WBC/BASO scattergram: This scattergram is obtained by lysing RBCs with a lysing reagent “STROMATOLYSER-FB” which selectively suppresses degranulation of basophils. Following this reaction, the sample is analysed by flow cytometry to detect forward and side scattered light information to give a WBC/BASO scattergram. The analyzer gives a total WBC count and basophil count based on this scattergram.^8^ Figure 1 shows a normal WBC-DIFF & WBC/BASO scattergram.

Various changes in the WBC scattergram (WBC-DIFF & WBC/BASO)like graying of WBC clusters, merging of clusters, multiple clustering, abnormal blue coded events and other changes if any, were analyzed simultaneously for all these cases.

Hematological parameters like hemoglobin (Hb), total leucocyte count (TLC), differential count (DC) and platelet count of all the malaria positive cases were collected.

Smears were reviewed for all the cases which showed abnormal scattergram. Sensitivity, specificity, Positive Predictive Value (PPV) and NegativePredictive Value (NPV) was calculated for both WBC-DIFF & WBC/BASO scattergrams using Galen and Gambino method.

## Results

During March 2013 to August 2013, we received 2610 peripheral smears for malarial parasite detection from patients who presented with fever, chills and rigors. Other uncommon presenting symptoms were vague abdominal pain, myalgia and headache. However, 2 cases presented with symptoms of Acute Renal Failure. Of the 2610 cases, 1730 were males and 880 were females with age ranging between 10–65 years.

Out of 2610 cases, 45 (n=45) cases were found to be malaria positive, of which 35 were males and 10 were females with age ranging from 15–65 years. Forty cases were positive for *Plasmodium vivax* and five cases were positive for *Plasmodium falciparum* with a *P.vivax* to *P.falciparum* ratio of 8:1.

**Figure 2:**
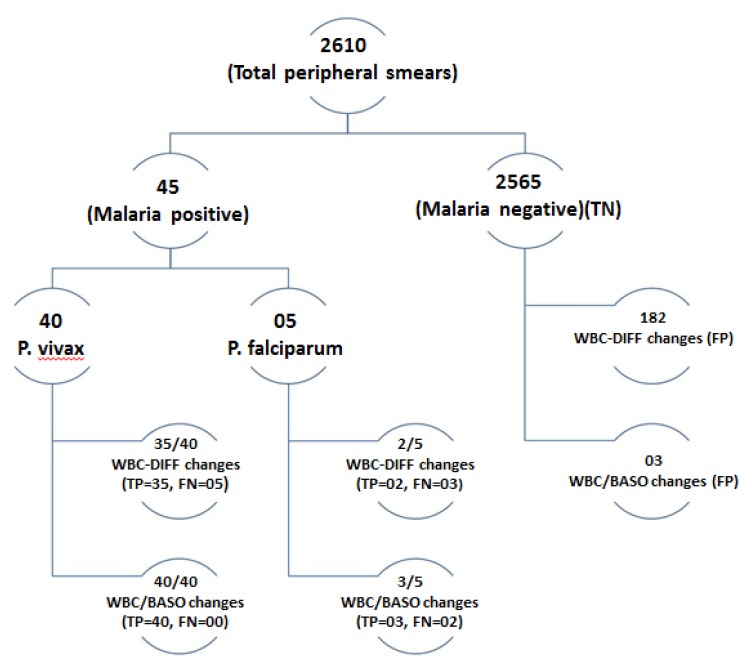
Distribution of cases. TP- True positive, TN- True negative, FP- False positive, FN- False negative.

Out of the 40 *P. vivax* cases, five cases were reported negative by initial peripheral smear examination, but these cases showed scattergram abnormalities and were diagnosed as positive on repeat peripheral smear. These missed cases were found to have very low parasite index. All the *P.falciparum* cases were diagnosed on peripheral smear examination. Total number of smear negative cases were 2565 during this period. Both WBC-DIFF and WBC/BASO scattergrams were analyzed in all the 2610 cases. Distribution of cases is given in figure 2.

### WBC-DIFF scattergram

WBC-DIFF abnormalities were found in 37 out of 45 malaria positive cases. In *P. vivax* group (n=40) 35 out of 40 cases showed various abnormities with a sensitivity of 87.5%. In *P. falciparum* group (n=5) 2 out of 5 cases showed WBC-DIFF abnormalities with a sensitivity of 40%. The overall specificity of WBC-DIFF plot in malarial detection was found to be 93.3%. PPV for *P. vivax* and *P. falciparum* was 16.13% and 1.09% respectively. NPV for both groups was found to be 99.8%. (Table 1)

**Figure 3:**
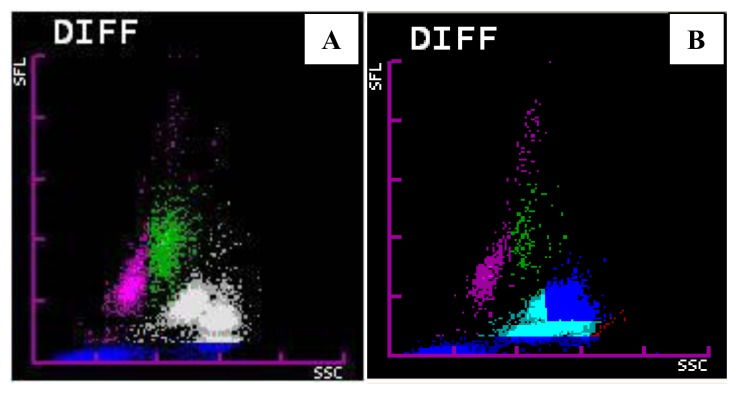
WBC-DIFF scattergram abnormalities. A: Graying of neutrophilic and eosinophilic clusters with blue coded events beneath. B: Merging of neutrophilic and eosinophilic clusters with blue coded events above the clusters.

Various changes noted in WBC-DIFF scattergram were merging of neutrophil and eosinophil clusters (44.5%), multiple neutrophil or eosinophil clusters (32%), graying of neutrophil and eosinophil clusters (27%), prominent blue coded events between/above or below the neutrophil and eosinophil clusters (22%) and large eosinophil clusters (10%) (Figure 3). These changes were noted singly or in combination.

WBC-DIFF abnormalities were also noted in 182 out of 2565 malaria negative cases. This included 95 new born blood samples, 46 cases of leukemia, 37 cases of other solid organ malignancies on chemotherapy and 4 cases of hemolytic anemia.

### WBC/BASO scattergram

**Figure 4:**
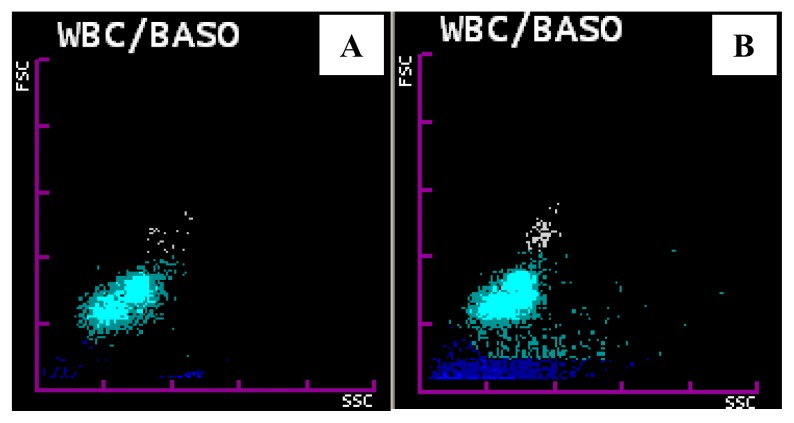
WBC/ BASO scattergram abnormalities. A: Showing prominent blue coded events in area III in a malaria positive case. B: Showing prominent blue coded events in late area II and area III in a malaria positive case.

WBC/BASO channel abnormalities were found in 43 out of 45 cases. In *P. vivax* group all the cases (n=40) showed one single most consistent change: isolated prominent blue coded events in area III or late area II and III in the WBC/BASO scattergram with a sensitivity of 100%.(Figure 4) In *P. falciparum* group, 3 out of 5 cases showed this change with a sensitivity of 60%. Overall specificity of WBC/BASO graph in malaria detection was found to be 98.9%. PPV for *P. vivax* and *P. falciparum* was 93.02% and 50% respectively. NPV for *P. vivax* and *P. falciparum* was 100% and 99.9% respectively. (Table 2)

**Figure 5A:**
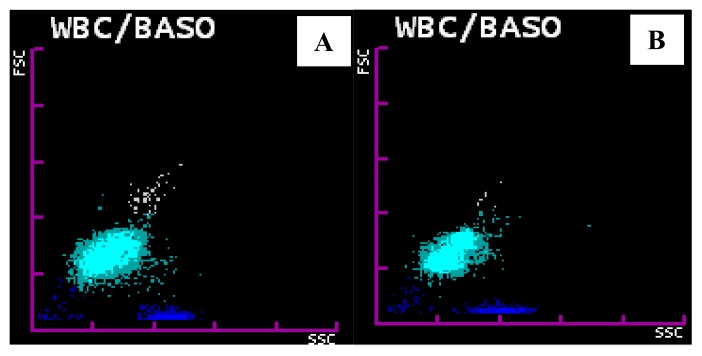
WBC/ BASO scattergram showing faint blue coded events in area III in a case of P. vivax which was missed on peripheral smear examination. **5B.** WBC/ BASO scattergram showing blue coded events extending from area I to III in a case of haemolytic disease of new born.

Of the 5 cases of *P. vivax* which were missed on peripheral smear, fine but faint blue coded dots ranging in number from 7 to 15 were found in area III of WBC/BASO channel (Figure 5A). This gave us the opportunity to review the smears and perform rapid card tests to give a final diagnosis of malaria.

Blue coded events were noted in 3 malaria negative cases out of which two cases had hemolytic disease of newborn and one was a case of thalassemia on treatment. These were confirmed to be malaria negative by a repeat smear and a negative card test. In these cases, in contrast to the malaria positive cases, the blue coding extended from area I to III (Figure 5B)

### Other findings

Thrombocytopenia was seen in 44 out of 45 malaria positive cases with platelet count ranging from 12–200 × 10^3^/μl with a mean of 53 × 10^3^/μl (SD 31.9). Pseudoeosinophilia which is defined as a difference in automated and manual eosinophil count of ≥5%, was noted in 7 out of 45 cases with a sensitivity of a mere 15.5%. It was also noted that all the scattergram abnormalities reversed after two days of initiation of antimalarial treatment.

## Discussion

In tropical and endemic countries where malaria is highly prevalent, there is a need for a rapid, cost effective and efficient method for screening the blood samples. Various new methods of malaria detection like quantitative buffy coat assay, antigen coated dipstick tests, rapid diagnostic card tests and polymerase chain reaction have come up in recent past.^9^,^10^ But these tests are limited by their high cost and limited feasibility. Due to these factors Romanowsky stained peripheral smear examination has remained as ‘gold standard’ in malaria diagnosis but the quality of malaria microscopy is far from satisfactory in most countries.^11^

There has been a growing interest in the use of automated hematology analyzers in the presumptive diagnosis of malaria.^12^ Earliest of such studies has been done by Hanscheid *et al.*^6^ on Cell Dyn3500 (Abott Diagnostics, USA), proving the efficacy of automated hematology analyzers in malaria detection using the principle of flow cytometry.

Sysmex XE-2100 & XT-2000i uses flow cytometry in conjunction with fluorescence properties of leucocytes to generate various WBC parameters, a few abnormalities of which can give a clue to the diagnosis of malaria.

Zuluaga *et al*.^13^ showed in their study that area III blue coded events in WBC/BASO scattergram for *P.vivax* had a sensitivity and specificity of 97% and 94% respectively using Sysmex XE-2100 autoanalyzer. For *P.falciparum* the sensitivity and specificity was found to be 60% and 67% respectively. These findings were similar to that of the present study.

Also, the presence of >8 blue coded events in area III of WBC/BASO plot when combined with the presence of thrombocytopenia increased the sensitivity of malaria detection. These findings are similar to the findings of Zuluaga *et al*.^13^

The WBC-DIFF plot abnormalities arise due to the neutrophils and eosinophils which have ingested the malarial pigment. WBC/BASO abnormalities are caused due to the red cells and reticulocytes which contain parasites and pigment.^12^

Yoo *et al.*^14^ reported in their study that psuedoeosinophilia and WBC scattergram abnormalities had sensitivity of 46.2% and specificity of 99.7%. Huh *et al.*^15^ also reported psuedoeosinophilia in 38% of malaria cases. In the present study psuedoeosinophilia was found in only 7 cases (15.5%). Psuedoeosinophilia is thought to be caused by the neutrophils which contain hemozoin pigment which are erroneously plotted in the eosinophil area. Pseudoeosinophilia was not a consistent finding in our cases, in contrary to previously reported studies and this could be due to a low parasitic index/load.

We also found 3 false positive cases in the WBC/BASO scattergram, but these cases showed blue coded events extending from area I to area III in contrast to isolated area III blue coded events in malaria positive cases. In our study, WBC scattergrams showed lower sensitivity and PPV in *P.falciparum* detection similar to studies conducted by Jain *et al.* [12] and Zuluaga*et al.*^13^ This could also be attributed to the low number of *P. falciparum* cases in the current study as *P.vivax* is the dominant malarial parasite in this part of the country.^16^ However, larger studies from *P.falciparum* predominant areas are required to further comment on the usefulness of analyzers in diagnosis of the same. In our study, abnormalities in WBC/BASO scattergram proved to be the most consistent finding in positive cases. Moreover, 5 cases of *P. vivax* which were missed on peripheral smear showed WBC/BASO plot abnormalities.

Thrombocytopenia was present in 97.7% of malaria positive cases in our study, which was similar to the findings by Abro *et al.* and Chandra *et al.*^17^,^18^ As the number of malaria cases presenting with isolated thrombocytopenia is very high, these cases should be specifically screened for malaria infestation.^19^

Last but not the least, this method was found to be the most cost effective diagnostic tool as CBC analysis costs 3–4 USD when compared to Malarial card test which costs nearly 8–10 USD in our setup.

## Conclusions

We found the WBC/BASO scattergram abnormalities to be useful in the presumptive diagnosis of *P. vivax* when combined with presence of thrombocytopenia. This helps the pathologists and technicians who handle these autoanalyzers to pick up all suspicious cases and subsequently confirm the same on a peripheral smear and with other rapid diagnostic tests.

## Figures and Tables

**Table 1:** Sensitivity, specificity, PPV and NPV for WBC-DIFF scattergram

	Sensitivity (%)	Specificity (%)	PPV (%)	NPV (%)
P. vivax	87.50	92.93	16.13	99.79
P. falciparum	40	92.93	1.09	99.87

Sensitivity = True positive**/**(True positive+False negative). Specificity = True negative**/**(True negative+False positive). Positive predictive value (PPV) = True positive**/**(True positive+False positive). Negative predictive value (NPV) = True negative**/**(True negative+False negative)

**Table 2:** Sensitivity, specificity, PPV and NPV for WBC/BASO scattergram

	Sensitivity (%)	Specificity (%)	PPV (%)	NPV (%)
***P. vivax***	100	99.88	93.02	100
***P. falciparum***	60	99.88	50	99.92

Sensitivity = True positive**/**(True positive+False negative). Specificity = True negative**/**(True negative+False positive). Positive predictive value (PPV) = True positive**/**(True positive+False positive). Negative predictive value (NPV) = True negative**/**(True negative+False negative)
